# flowCore: a Bioconductor package for high throughput flow cytometry

**DOI:** 10.1186/1471-2105-10-106

**Published:** 2009-04-09

**Authors:** Florian Hahne, Nolwenn LeMeur, Ryan R Brinkman, Byron Ellis, Perry Haaland, Deepayan Sarkar, Josef Spidlen, Errol Strain, Robert Gentleman

**Affiliations:** 1 Life Sciences Department, Computational Biology Program, Division of Public Health Sciences, Fred Hutchinson Cancer Research Center, 1100 Fairview Ave N, M2-B876, PO Box 19024, Seattle, Washington 98109-1024, USA; 2 EA SeRAIC INSERM, IRISA – Symbiose, Campus Beaulieu, Université de Rennes I, 35042 Rennes Cedex, France; 3 Terry Fox Laboratory, British Columbia Cancer Agency Research Centre, 675 West 10th Avenue, Vancouver, BC V5Z 1L3, Canada; 4 AdBrite Inc, 731 Market St, 5th Floor, San Francisco, California 94103, USA; 5 BD Biosciences, Research Triangle Park, North Carolina 27709, USA

## Abstract

**Background:**

Recent advances in automation technologies have enabled the use of flow cytometry for high throughput screening, generating large complex data sets often in clinical trials or drug discovery settings. However, data management and data analysis methods have not advanced sufficiently far from the initial small-scale studies to support modeling in the presence of multiple covariates.

**Results:**

We developed a set of flexible open source computational tools in the R package flowCore to facilitate the analysis of these complex data. A key component of which is having suitable data structures that support the application of similar operations to a collection of samples or a clinical cohort. In addition, our software constitutes a shared and extensible research platform that enables collaboration between bioinformaticians, computer scientists, statisticians, biologists and clinicians. This platform will foster the development of novel analytic methods for flow cytometry.

**Conclusion:**

The software has been applied in the analysis of various data sets and its data structures have proven to be highly efficient in capturing and organizing the analytic work flow. Finally, a number of additional Bioconductor packages successfully build on the infrastructure provided by flowCore, open new avenues for flow data analysis.

## Background

Automation technologies developed during the last several years have enabled the use of flow cytometry (FCM) to generate large, complex data sets in both basic and clinical research applications [1]. A serious bottleneck in the interpretation of existing studies and the application of high throughput FCM to even larger, more complex problems is that data management and data analysis methods have not advanced sufficiently far from the methods developed for applications of FCM to small-scale, tube-based studies [2]. In particular, the data often need to be organized into groups of samples based on combinations of additional covariates and similar operations need to be applied to these groups in a transparent and reproducible manner. Furthermore, the growing depth of knowledge in the field of immunology, for instance the characterization of distinct human T-cell sub-population [3], clearly argues for more systematic approaches.

Some of the consequences of the lag of efficient software solutions are difficulties in maintaining the integrity and documentation of large data sets, assessing measurement quality, developing validated assays, controlling the accuracy of gating techniques, automating complex gating strategies, and aggregating statistical results across large study sets for further analysis. In addition, new analysis approaches face difficulty in finding their way into standard practice. We believe that these barriers to the development and dissemination of new analysis methods is one of the fundamental restraints on the future expansion of FC-HCS in both clinical and research applications.

Traditionally, for the majority, FCM experiments were being analyzed by manual data inspection in one or two dimensions, or by very basic comparisons of summary statistics. Most of the currently available analysis tools are designed to reflect this work flow. We believe that these approaches, in addition to being expensive and labor intensive, do not fully address the highly complex nature of FCM data; in particular, they disregard many of the fundamental aspects of the data, such as sample groups or cohorts, the underlying distribution or its high-dimensional nature. Furthermore, the subjective character of manual analyses are a major obstacle to reproducibility. In a recent study of flow cytometric standardization involving 15 institutions, the mean inter-laboratory coefficient of variation ranged from 17 to 44%, even though preparation was standardized and performed using the same samples and reagents at each site [4]. For FC-HCS data, unassisted manual inspection is extremely time consuming, and robust statistical methods need to be developed to point investigators to interesting aspects of the data, or to potential problems. While the expert knowledge of immunologists and researchers remains crucial for the understanding of FCM data, we believe that collaboration with other research fields such as statistics and computer science can greatly improve the relevance of FCM in today's high-throughput paradigm. In this paper, we describe a set of flexible and well structured computational tools to efficiently analyze FC-HCS data. Our intent is to provide a shared research platform that enables bioinformaticians, computer scientists, and statisticians to work collaboratively with biologists and clinicians to develop novel methods for FCM data analysis, a process deemed crucial by many for the further development of the technology [5].

## Implementation

The computational tools we have developed are distributed in the R software language [6] as the Bioconductor [7] package flowCore. The package flowCore is a freely available, highly functional, and extensible FCM data analysis platform that enables researchers to efficiently handle FC-HCS data and encourages open development of tools for their coherent analysis. In our implementation of flowCore we rely on two important lessons learned from the field of gene expression data analysis: the first being the importance of data structures that reflect the underlying data and facilitate the manipulations that are of most interest, while the second is the importance of a modular architecture that allows for many developers to extend and use the underlying infrastructure and to combine tools in complex work flows. flowCore implements such computationally efficient data structures and a range of specialized methods addressing all components of a typical FCM analysis work flow, including compensation, transformation, and gating. flowCore runs on Windows, Mac OS X, and Linux/Unix operating systems.

### Existing data standards and conventions

Currently, data from FCM experiments are stored in single files according to the Flow Cytometry Standard (FCS) [8]. However, recent developments in high-throughput FCM are shifting the focus of interest away from single-tube based measurements towards large and complex experimental designs with dozens of covariates and influencing factors. For example, experiments consist of large numbers of samples from different patients, measured at different time points [1] or following different drug treatments [9]. Modern FCM data analysis tools have to deal with an additional layer of sample metadata and they need to provide infrastructure to process and to compare groups of samples in a concise and coordinated manner. The notion of classes from an object-oriented programming language provides one coherent way to describe these richer data structures. In addition, functions or methods that work on those classes allow for interaction and manipulation. Many of the currently available software solutions offer only limited support for such self-contained structures, or make use of binary storage containers that are designed specifically for the needs of particular user interfaces and hence are not easily amenable to programmatical access. In addition, the closed-source nature of these products often makes them impractical to integrate into analysis pipelines. In this manuscript we describe classes for FCM data analysis and their implementation in R, however, they could just as easily be implemented in any other language (e.g., Java, C++). Software written in those languages could use similar data structures, thereby simplifying communication and the interchange of data between analysis tools.

flowCore does not provide a graphical user interface and all operations are done using a command line interface. It is possible to add a more elaborate user interface on top of this infrastructure, however the focus in this paper is on a programmatic approach to enable the convenient development of novel analysis methods and automation of complex analysis approaches. By taking the burden of data management from the programmer, and by providing well-defined application programming interfaces (APIs), it is possible to readily test new ideas and to easily extend the framework's functionality.

**Figure 1:**
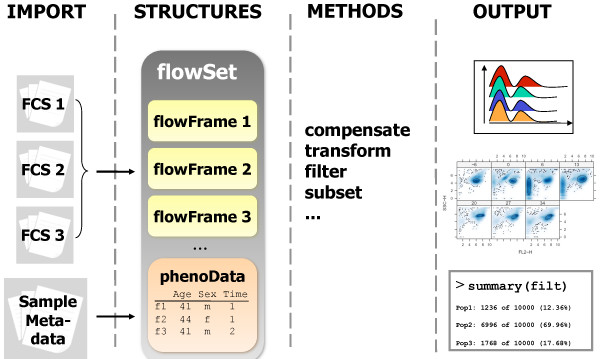
**flowCore framework**. For each experiment, the content of the FCS files, phenotypic and metadata are stored in a *flowSet*. Each *flowFrame *in a *flowSet *corresponds to one FCS file. All basic operations (e.g., compensation, transformation, gating) can be applied to either single *flowFrames *or a *flowSet *simultaneously.

The flowCore framework presented here can import and process raw data FCS files along with their complete set of file-specific metadata (Figure 1). Moreover, it is a software implementation of the Gating Markup Language Candidate Recommendation, an emerging standard developed in collaboration with the International Society for Analytical Cytology (ISAC) Data Standards Task Force, which makes it possible to integrate flowCore in existing work flows and to communicate with any other FCM tool that adheres to the proposed standard [10]. Adherence to standards also plays a critical role in the ability of new methods based on flowCore to find their way back into the standard practices for FCM data analysis.

### Basic Data Structures

#### flowFrame: sample unit

flowCore's primary task is the representation and basic manipulation of FCM data. This is accomplished through a data model very close to that adopted by other successful Bioconductor packages. All information from a single FCS file, *i.e.*, the collection of events and the accompanying metadata, is stored in one single container. We call the structure that hold this data a *flowFrame *(Figure 1). Raw data values as well as associated metadata of a *flowFrame *can be accessed programmatically. Most commonly, the metadata consist of descriptors of the stains used in the experiment and the respective measurement channels, information about compensation performed at the instrument side and any additional keywords the user deems to be important to annotate the data. During the creation of a *flowFrame*, a number of quality checks are performed to ensure data integrity.

#### flowSet: a collection of flowFrames

In high-throughput FCM, many of the analysis tasks need to be performed consistently across multiple samples, hence we introduce the concept of a collection of *flowFrames *called a *flowSet *(Figure 1). A *flowSet *is a container for multiple *flowFrames *along with relevant information associated with each individual frame such as descriptions of the cell sample, the treatment to which the sample was subjected, or the location of that sample in a microtitre plate. The objects are self-contained and can be shipped to other computers, platform independently. *flowSets *manage the consistent application of operations on the individual *flowFrames *and shift the burden of keeping score of the metadata from the user to the infrastructure, thus reducing the risk of errors (*e.g.*, mixups of sample labels). Crucial operations like taking subsets, data transformations and gating, or computation of summary statistics are greatly facilitated and all relevant annotation information is constantly passed on along the analysis pipeline. The *flowSet *structure can be readily extended to incorporate the potentially complex metadata associated with even larger FC-HCS experiments such as clinical trials, where hundreds of patients might provide samples at different time points over the course of the experiment. The *flowSet *data structure is one of the key features in the flowCore package and it is fundamental to the implementation of many of the high level functionalities such as quality assessment and control, visualization and automated gating.

### Standard flow operations

Typically, the basic operations in FCM analyses adhere to the following common work flow: the data need to be compensated (if that was not already done on the instrument) and transformed, and sub-populations of interest need to be selected based on a set of (predominantly sequential) gates. All software solutions for FCM analysis offer support for these operations, most often in an interactive, graphical user interface. In flowCore we have taken the approach to abstractly describe these operations and build a set of tools to perform them on both *flowFrames *and *flowSets*. Typically, the results of these operations are again *flowFrame *or *flowSet *objects. While transformation, and to a certain extent compensation, are fairly routine operations with only limited potential for improvement, being able to implement new methodologies for gating of FCM data, and extend the capabilities of flowCore through object oriented programming are features that clearly sets our framework apart from other FCM analysis tools. By factoring out as much of the bookkeeping as possible, programmers can focus on the actual operations rather then having to deal with the tedious details of data integration and access. Third-party methods can act on their own as first-class citizens in the analysis framework, without breaking the work flow or the basic infrastructure. This design allows for the straightforward extension of flowCore's capabilities, and has already fostered the development of a number of valuable add-ons [11,12].

#### Transformation and compensation

Data transformation is essential for both data visualization and modeling [11]. The major transformations that are routinely used in FCM analysis have been implemented in flowCore (*e.g.*, log, bi-exponential, arcsinh or logicle [13], see Table 1 for a complete list). Furthermore, the design of the R language makes it easy to define arbitrary functions to apply to the data of individual *flowFrames *or entire *flowSets*, respectively. Compensation, that corrects for fluorescence spillover originating from the inherent overlap of emission spectra from antibody fluorescent labels, is available for both *flowFrames *and *flowSets*. In addition, the software offers functionality to compute spillover or compensation matrices from a set of appropriate single stain controls.

**Table 1:** Data transformations implemented in flowCore.

	Data Transformations
linear	*ax *+ *b*
quadratic	*ax*^2 ^+ *bx *+ *c*
natural logarithm	*log*_*e*_(*x*)(*r/d*)
logarithm	*log*_*b*_(*x*)(*r/d*)
biexponential	*ae*^(b*x) ^– *ce*^(-*d*+*x*) ^+*f*
logicle	*Te*^-(*m*-*w*)^(*e*^(*x*-*w*)^-*p*^2^*e*^-(*x*-*w*)/*p *^+ *p*^2^-1)
truncate	*x*_*x*≤*a *_= *a*
scale	(*x-a*)/(*b-a*)
arcsinh	*arcsinh*(*a *+*bx*) + *c*

Within these formulas, *x *is the variable corresponding to value being transformed, *a*, *b*, *c*, *d*, *f*, *p*, *m*, *T*, and *w*, are constants affecting the transformation function, *e *is the base of the natural logarithm (see [13] for details on the logicle transformation). Other transformations can easily be implemented in R.

#### Gating

In flowCore, gating operations are represented by classes that can be extended in an object-oriented manner (Table 2). Basic gate types such as rectangular gates, ellipses and polygon gates are implemented as part of the framework. In addition, we introduce the notion of data-driven gates, or filters, for which the necessary parameters are computed based on the properties of the underlying data, for instance by modeling data distribution or by density estimation. This approach is fundamentally different from the traditional application of static gating regions across samples, as it is able to take into accounts unforeseen changes in signal intensities, such as drifts in the instrumentation over time or sample variability.

**Table 2:** Filter and gate classes implemented in flowCore.

	Gates
rectangleGate	n-dimensional rectangular regions
quadGate	quadrant regions in two dimensions
polygonGate	polygonal regions in two dimensions
polytopeGate	generalization of polygon in n dimensions
ellipsoidGate	n-dimensional ellipsoid region

	Filters

sampleFilter	random sub-sampling
expressionFilter	results of a boolean expression
kmeansFilter	K-means clustering
norm2Filter	bivariate normal distribution
curv1Filter	local density regions in 1D
curv2Filter	density regions in 2D
timeFilter	abnormal data acquisition over time

filterSet	gating strategies

Filters are automated, data driven procedures. Gates are static, user-defined methods.

The ability to programmatically access gates is a prerequisite for semi-automated or automated gating. By utilizing an unified interface for all different types of gates, the user is able to subset data sets as well as to create summary statistics, as for instance the proportions of events falling in a single gate or in a combination of gates. Complex combinations and hierarchies of gates can be captured in multi-step gating strategies. The definition of gates in flowCore follows the Gating Markup Language Candidate Recommendation [10], thus any flowCore gating strategy can be reproduced by any other software that also adheres to the standard and *vice versa*.

Gating, as well as all other operations in *flowCore*, can be applied over each individual frame in a *flowSet*, and summary methods provide information about the outcome of these operations. In addition, the result of a gating operation can be used to subset the input *flowFrame *or *flowSet*, either by filtering out negative events or by splitting in multiple sub-populations. This design allows to easily combine all of flowCore's components into complex work flows.

## Results and discussion

### From quality assessment to batch gating

The flowCore package has been successfully applied in the analysis of several data sets, both originating from clinical trials [1] and drug discovery experiments [9]. A complete description of the methods implemented in flowCore is beyond the scope of this publication. Much more comprehensive documentation and users guide information with programmatic examples are available online , as part of the package distribution. Here, we want to briefly exemplify some of the software's key features, that is, the coherent treatment of all samples in a potentially large experiment, the concept of data-driven automated gating, the integration of existing software into the framework, and the generation of publication-quality graphics for data visualization.

**Figure 2:**
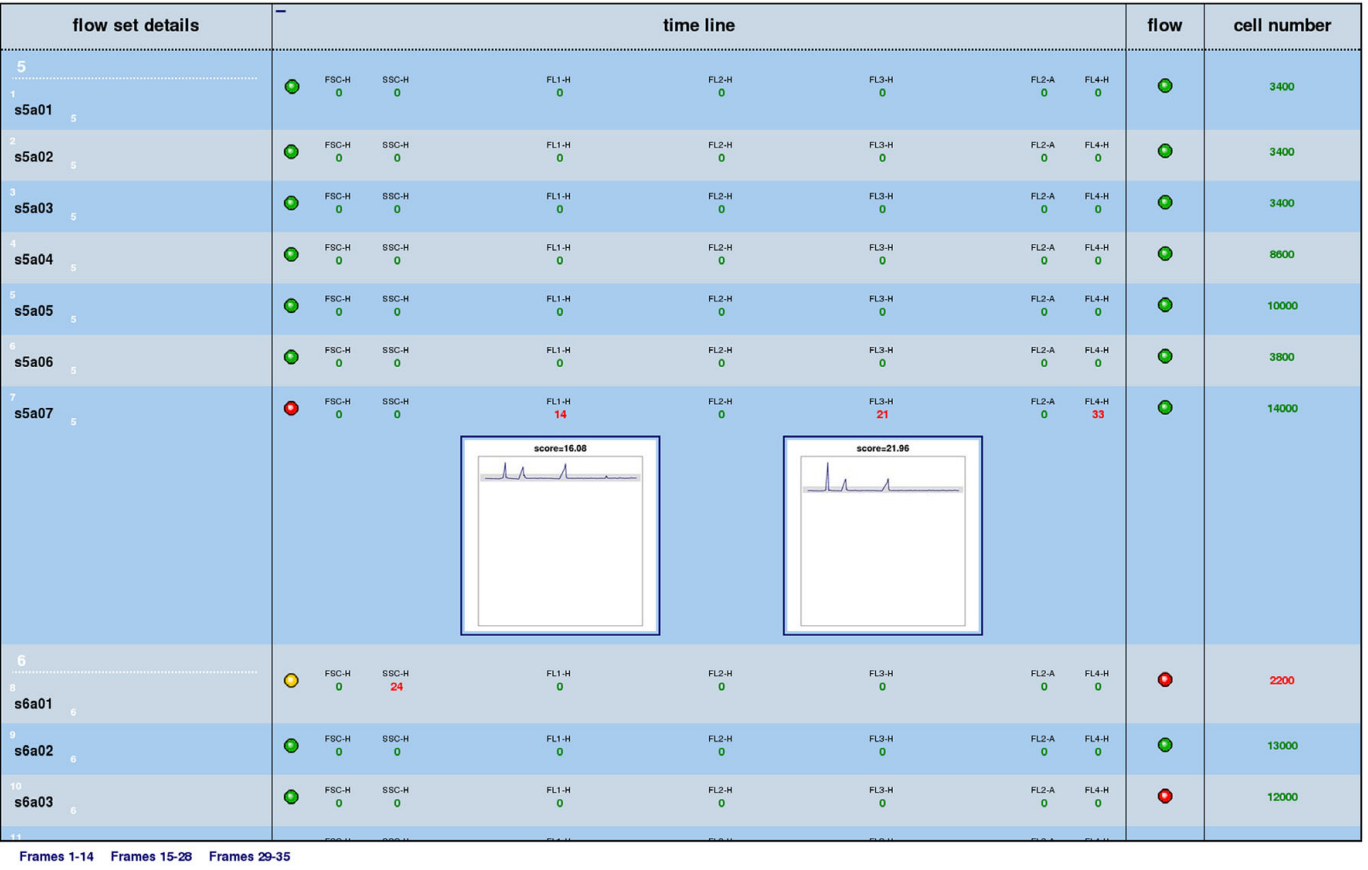
**Quality assessment**. HTML quality assessment report generated by the flowQ package for a subset of data from an experiment focusing on Graft-Versus-Host Disease [1]. Rows correspond to the samples in the set, columns to different quality checks.

Data analysis for most experiments usually begins with a quality assurance step. In a FCS analysis work flow, we can use functionality from the flowQ package, build upon flowCore, to create an HTML report that highlights potential quality issues. Assuming that the data has already been imported as the *flowSet *object "dat", using for instance the *read. flowSet *method, the following simple lines of code produce the output shown in Figure 2:

> library(flowQ)

> qaReport(dat, c("qaProcess.timeline",

                           "qaProcess.timeflow",

                            "qaProcess.cellnumber"))

The report is interactive and provides drill-down to more detailed aspects of the analysis, starting from a concise overview. The design of flowCore's data model allows for a coherent treatment of all the samples, hence we are able to compare features between individuals, or between groups of individuals, based on the available metadata information.

According quality of the measurements, the next steps of a FCS analysis work flow are potentially the compensation and transformation of the data. Once again flowCore's data structure and its methods allow a flexible processing. One can use the basic transformation and compensation functions implemented in flowCore (Table 1) or develop its own approaches that could then be apply to *flowFrame *or *flowSet*. Ultimately, a flow cytometry experiment aims at identifying and characterizing cell population of biological interest, using static gating or data-driven procedures (Table 2). Static gating for all samples in a high-throughput FCM experiment is often impossible, since the measured variables tend to vary between different treatments, over time or between different experiment batches. Automated or data-driven gating has the potential to estimate the gating regions from the underlying data, thus providing a fast objective solution to the analysis of potentially very large and diverse data sets [11]. One of the automated gating methods implemented in flowCore is based on identifying areas of significant curvature in a kernel density estimate of the data [14]. Assuming that the regions of interest are of high density, the software is able to reliably detect them in a one- or two-dimensional density landscape.

> cf <- curv2Filter("FL1-H", "FL3-H")

> fres <- filter(dat, cf)

Kernel density estimation is a well-known problem in statistical computing, and a lot of effort has been invested in the development of good software to address it. The modular design of flowCore allows to easily integrate these existing solutions into our framework. In this example, we directly use R code from the feature package [14]. Instead of re-writing existing code, we are able to include it via the well tested distribution mechanism provided by R's software package system. This process is bi-directional, and all functionalities implemented in flowCore are available to other package authors.

**Figure 3:**
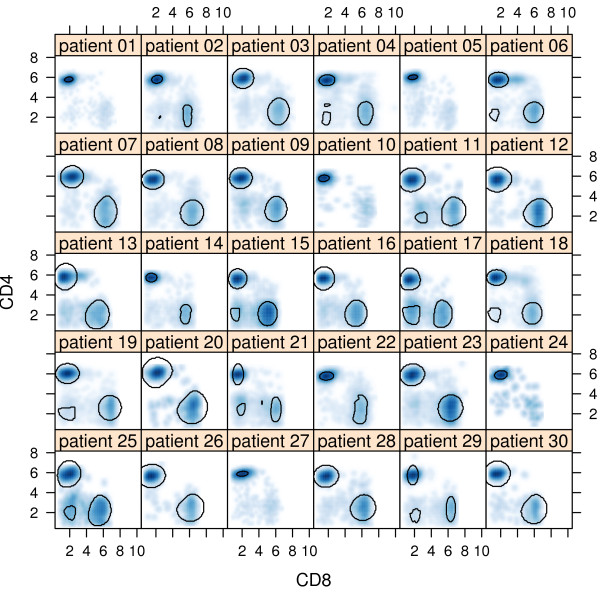
**Batch gating**. Scatterplot matrix of a single *flowSet *from an experiment focusing on immune tolerance following kidney transplantation. Outlines of the gating regions identified by a *curve2Filter *automated gating operation are added on top of the density representation of the data.

Finally, we can chose one of the many visualization options from the flowViz package to plot the results of the recent filtering operation. A very basic matrix of density plots is shown in Figure 3, where each panel in the matrix represents the fluorescent measurements of two channels for one individual patient.

> xyplot('FL1-H' ~ 'FL3-H' | SampleID,

            data = dat, filter = fres)

Scripting languages like R provide a natural representation of work flows through a sequence of code instructions in regular text files. This allows for the rapid development and testing of new ideas, however it is not very well suited for routine data analysis tasks. Furthermore, the overhead of data management and variable tracking can be considerable. To that end, flowCore also provides data structures that help organize sequences of typical FCM data analysis operations and complex gating strategies into concise work flows. These structures are self-re effective, they contain all intermediate results and offer a unified user interface to assess the progress and the outcome of an analysis.

### Related flow packages

In addition to the flowCore package that offers basic infrastructure, we have implemented a range of additional Bioconductor packages that are dedicated to more specific tasks of FCM data analysis. As exemplified in the previous section, the flowViz package [12] provides sophisticated data visualization tools, that make use off multivariate trellis plotting [15]. These functions can be used to quickly generate customized plots for extended cytometry data sets for both direct data inspection and quality control. The objects metadata information can be used to arrange the layout and composition of the plots.

Furthermore, the design and the API of the visualization software is very generic, and users can readily extend its capabilities by providing self-defined plotting functions. The flowQ package offers more advanced quality assurance methodology and a framework to create interactive web-based reports of quality assurance results. The flowUtil package implements data import and export including flow-cytometry specific standard markup language. Finally, the flowStats package provides elaborate statistical methods that are relevant in the context of flow cytometry data analysis.

More recently, [11] have developed an automatic gating approach via robust model-based clustering using flowCore's data model and infrastructure which is implemented in the Bioconductor package flowClust. Another package, plateCore, providing more specialized support for experiments conducted on microtitre plates and facilitating the handling of spatial metadata, is under development.

## Conclusion

Through flowCore, we have provided the FCM community with an open source, freely available, highly functional, and standards compliant, development and analysis platform for high throughput data analysis. We hope to foster collaborative development of new analysis methods and to facilitate the transition of these new methods to a larger flow community. Our experience has been that such collaborative effort has proven beneficial for a number of different biological and computational biology challenges, greatly elevating their applicability. We hope that our framework will be the foundation for fruitful shared research by many collaborators from multiple scientific fields and will help resolve bottlenecks that currently prevent further development and deployment of FC-HCS to increasingly complex and important scientific and clinical applications.

## Availability and requirements

Project name: flowCore; Project home page: ; Operating system(s): A wide variety of UNIX platforms, Windows and MacOS.; Programming language: R; License: The Artistic License, Version 2.0.

The flowCore package and its associated packages are part of the R/Bioconductor project, an environment for statistical computing and bioinformatics. The R software environment is freely available at . flowCore and its dependencies (flowQ, flowViz) are available on the Bioconductor project website  as freely distributed and open source software packages with an Artistic license. They are fully integrated into the R/Bioconductor environment for statistical computing and bioinformatics and run on operating systems Windows, Mac OS X, and Unix.

## Authors' contributions

The project was conceived by RB, RG, PH. It was designed, and developed by all the authors. NLM, FH, DS, and BE wrote most of the software's code. The manuscript was prepared by FH and NLM, then revised and approved by all authors.
